# Child and parent predictors of picky eating from preschool to school age

**DOI:** 10.1186/s12966-017-0542-7

**Published:** 2017-07-06

**Authors:** Silje Steinsbekk, Arielle Bonneville-Roussy, Alison Fildes, Clare H. Llewellyn, Lars Wichstrøm

**Affiliations:** 1 Department of Psychology, Norwegian University of Science and Technology (NTNU), Dragvoll, 7491, Trondheim, Norway; 2 Grove House, Roehampton Lane, Roehampton University, London, SW15 5PJ UK; 3 School of Psychology, University of Leeds, Leeds, LS2 9JT UK; 4 Department of Behavioural Science & Health, University College London, 1-19 Torrington Place, London, WC1E 7HB UK; 5 NTNU Social Research, 7491 Trondheim, Norway

**Keywords:** Picky eating, Pickiness, Fussiness, Sensory sensitivity, Parenting, Structuring, Sensitivity, Temperament

## Abstract

**Background:**

Picky eating is prevalent in childhood. Because pickiness concerns parents and is associated with nutrient deficiency and psychological problems, the antecedents of pickiness need to be identified. We propose an etiological model of picky eating involving child temperament, sensory sensitivity and parent-child interaction.

**Methods:**

Two cohorts of 4-year olds (born 2003 or 2004) in Trondheim, Norway were invited to participate (97.2% attendance; 82.0% consent rate, *n* = 2475) and a screen-stratified subsample of 1250 children was recruited. We interviewed 997 parents about their child’s pickiness and sensory sensitivity using the Preschool Age Psychiatric Assessment (PAPA). Two years later, 795 of the parents completed the interview. The Children’s Behavior Questionnaire (CBQ) was used to assess children’s temperament. Parent- child interactions were videotaped and parental sensitivity (i.e., parental awareness and appropriate responsiveness to children’s verbal and nonverbal cues) and structuring were rated using the Emotional Availability Scales (EAS).

**Results:**

At both measurement times, 26% of the children were categorized as picky eaters. Pickiness was moderately stable from preschool to school age (OR = 5.92, CI = 3.95, 8.86), and about half of those who displayed pickiness at age 4 were also picky eaters two years later. While accounting for pickiness at age 4, sensory sensitivity at age 4 predicted pickiness at age 6 (OR = 1.25, CI = 1.08, 2.23), whereas temperamental surgency (OR = 0.88, CI = 0.64, 1.22) and negative affectivity (OR = 1.17, CI = 0.75, 1.84) did not. Parental structuring was found to reduce the risk of children’s picky eating two years later (OR = 0.90, CI = 0.82, 0.99), whereas parental sensitivity increased the odds for pickiness (OR = 1.10, CI = 1.00, 1.21).

**Conclusions:**

Although pickiness is stable from preschool to school age, children who are more sensory sensitive are at higher risk for pickiness two years later, as are children whose parents display relatively higher levels of sensitivity and lower levels of structuring. Our findings suggest that interventions targeting children’s sensory sensitivity, as well as parental sensitivity and structuring, might reduce the risk of childhood pickiness. Health care providers should support parents of picky eaters in repeatedly offering unfamiliar and rejected foods to their children without pressure and acknowledging child autonomy.

**Electronic supplementary material:**

The online version of this article (doi:10.1186/s12966-017-0542-7) contains supplementary material, which is available to authorized users.

## Background

The unwillingness to eat certain familiar or unfamiliar types of food, known as picky eating [1], is most prevalent in early childhood, with rates ranging from 5.6 to 59.3% depending on the definition and assessment methods used [2]. Pickiness is of great concern for parents [3]. Although knowledge of the health-related outcomes of picky eating is limited due to a lack of longitudinal studies [2], research suggests that picky eating is associated with nutrient deficiency [4], underweight [5], behavioral problems [6] and symptoms of anxiety and depression [7]. Even though pickiness may decrease somewhat during the late preschool and early school years [2], evidence suggests a substantial number of children continue to be picky into school age [3], whereas others first display picky eating after starting school [8]. Further, pickiness also seems to be more persistent with later onset, while early onset cases recover more quickly [3]. Therefore, identifying the predictors of pickiness in the period of transition from preschool to school would be especially valuable.

Two longitudinal studies have examined socio-demographic predictors of picky eating [8, 9], and one investigation focused on parental negativity [10]. However, research into modifiable risk factors (e.g., breastfeeding, parenting) has been cross sectional, limiting the possibility of causal inferences between those risk factors and picky eating [11]. Children’s eating is influenced by their dispositional qualities such as temperament [12–14], but also by parenting practices and the way parents feed and react to their child’s eating [15, 16]. We therefore propose an etiological model of picky eating involving child temperament, sensory sensitivity and parent-child interaction. We test this model in a large and representative sample of Norwegian children followed from 4 to 6 years of age.

### Temperament, sensory sensitivity and pickiness

Individual differences in temperament may explain why some children develop eating problems whereas others do not [12, 17]. According to Rothbart’s work, three overarching factors of temperament can be found: Surgency/extraversion, negative affectivity and effortful control [18]. Surgency, the tendency to be approached-oriented and sensation-seeking, may cause children to be more open to new food experiences. Studies conducted with adults show sensation seeking to be negatively associated with food neophobia, i.e. the avoidance of novel foods, which is closely related to pickiness [19–21]. Notably though, Hafstad et al. [10] did not find that level of sociability (akin to surgency) predicted decreased pickiness in very young children (1.5 to 4.5 years of age), but it is not known whether this also applies to older children. Negative affectivity, which is characterized by mood instability, angry reactivity and dysregulated negative emotions [22, 23], is also associated with picky eating in cross-sectional [9, 12], and prospective studies [10]. We therefore examine both surgency and negative affectivity as predictors of pickiness from preschool to school age.

Effortful control, the third overarching temperamental dimension comprising inhibition and planning [18], does not intuitively appear relevant to picky eating. However, perceptual sensitivity, one of its sub dimensions may be pertinent. Perceptual or sensory sensitivity is conceptualized as low neurological thresholds for responding to sensory events and passive response strategies [24]. Individuals high in sensory sensitivity not only notice *more* sensory events (from taste, touch, vision, and smell) than others, but they notice sensory stimuli, such as food textures, more *rapidly* [24]. As the sensory properties of foods vary, it is reasonable to assume that sensory sensitivity will affect food acceptance [25]. In fact, sensory sensitive children have been found to be more reluctant to try new foods (food neophobia), eat fewer fruits and vegetables [26], and display higher levels of pickiness in cross-sectional studies [7, 27]. However, it is not yet clear whether sensory sensitivity *prospectively* predicts picky eating.

### Parenting as a predictor of pickiness

Simply stated, picky eating is characterized by one defining behavior; avoidance of food. Thus, picky eating manifests itself through avoidant behavior, as in anxiety disorders, and may similarly persist by means of negative reinforcement. Parents are concerned about picky eating [3] but may respond to this behavior differently. We propose two parenting pathways that potentially drive pickiness in early childhood, described below.

### Protective pathway: parental structuring

Parents who adopt a structuring/scaffolding parenting style, who teach and help the child while acknowledging the child’s autonomy [28], may offer their child unfamiliar or disliked foods in a gentle, yet firm way, encouraging the child to try. This parental approach is comparable to an authoritative feeding style, characterized by emotional warmth and responsiveness as well as high dietary expectations [29]. Indeed, authoritative parenting (i.e. high involvement and high control) is associated with healthy eating behavior [16], including higher fruit and vegetable consumption [29], and has been shown to reduce the negative association between pickiness and fruit intake [30]. Parenting behavior characterized by non-aversive, reinforcing parent-child-interactions, the caregiver being proactive and structuring, has further shown to prospectively predict children’s dietary quality [31]. In sum, research supports a link between general parenting and dietary practices [32, 33], structuring and autonomy support being considered two of the most important parenting constructs as regards parent’s impact on children’s diet and eating habits [34]. Although relatively little is known about the management of pickiness [11], repeated exposure to a novel and/or disliked food has been shown to increase children’s acceptance of the food [35]. However, in order for exposure interventions to be effective, caregivers have to be systematic in their effort to familiarize their child with foods, for instance, by repeatedly and methodically offering certain rejected foods on multiple occasions and praising or rewarding the child for tasting [36, 37]. Such behavior requires structuring of the child’s environment and scaffolding the child’s learning. We therefore expect high parental structuring/scaffolding to reduce the risk of the child becoming a picky eater, whereas little or no structure and scaffolding is expected to increase the risk of prospective pickiness.

### Risk pathway: parental sensitivity

A child’s negative response to food can be distressing for parents [3]; however, some parents may be more distressed than others. Parental sensitivity, defined as awareness and appropriate responsiveness to children’s verbal and nonverbal cues, is generally thought to be psychologically beneficial [38]. However, high sensitivity may have its drawbacks. Because the picky child needs to endure uneasiness, or even anxiety to try a rejected food, the parent similarly needs to endure their child’s uneasiness. Highly sensitive parents may be more affected by their child’s distress and therefore more inclined to avoid confrontation or to abort efforts to encourage their child to try new foods when s/he protests or becomes distressed. This is the proposed *sensitive pathway* to picky eating, whereby high parental sensitivity leads to decreased food exposure, and thereby negatively reinforces the child’s avoidance. Of note, highly sensitive parents also tend to be structuring [39]. Therefore, we do not expect structuring and sensitivity, which we hypothesize to work in opposite ways, to be bivariately associated with pickiness, because these opposite effects may cancel each other out. However, when adjusted for each other, the ‘true colors’ of structuring and sensitivity may appear.

### Current study

Pickiness is associated with several negative physical and psychological health outcomes and can cause parental distress. Identification of predictors is pertinent to inform interventions aimed at reducing picky eating in childhood. We aim to extend existing research by examining child- and parenting factors as predictors of pickiness from age 4 to 6. Specifically, we will explore child temperament, sensory sensitivity, parenting sensitivity and structuring as predictors of pickiness. This study extends earlier findings by applying a psychiatric interview to capture pickiness rather than single items or questionnaires used in earlier cross-sectional [2] and longitudinal studies [8, 10]. We hypothesize that pickiness at age 6, adjusted for pickiness at age 4, would be predicted by: 1) higher levels of child negative affectivity and lower levels of surgency; 2) higher levels of sensory sensitivity; and 3) greater parental sensitivity (i.e., the ability to effectively read and act upon the child’s cues) and lower levels of parental structuring. Gender differences are also explored.

## Methods

### Participants and procedure

A letter of invitation to participate in the study, together with the Strengths and Difficulties Questionnaire (SDQ) 4–16 version [40], was sent to the homes of all children born in 2003 and 2004 living in Trondheim, Norway (*N* = 3456). The parents brought the completed SDQ when attending the regular health check-up for 4-year olds, where a health nurse informed parents about the study and asked them to participate (*n* = 3016). Of the eligible parents, 97.2% (*n* = 3358) met for the appointed health check-up, 2475 gave informed consent, and 1250 children were drawn to participate. Children were allocated to four strata according to their SDQ scores to oversample for mental health problems (cut-offs: 0–4, 5–8, 9–11, and 12–40) and the probability of selection increased with increasing SDQ scores (.37, .48, .70, and .89 in the four strata, respectively). The sample is comparable to the Norwegian parent population for the parents’ level of education [41]. We succeeded in interviewing parents of 997 children at Time 1 (T1), when the children’s mean age was 4.7 years (SD = 0.30). At follow up two years later, 795 children participated (mean age = 6.7 years, SD = 0.17). Further details of the recruitment procedure is presented in Wichstrøm et al. [42]. All procedures were approved by the Regional Committee for Medical and Health Research Ethics Mid Norway.

### Measures

#### Pickiness

A semi-structured psychiatric interview, The Preschool Age Psychiatric Assessment (PAPA) [43], was used to assess children’s picky eating at both measurement points. The PAPA assesses symptoms of psychiatric disorders in preschool children, but also includes items related to picky eating. More specifically, parents are interviewed about their child’s food preferences and appetite over the last three months, whether the child consumes only restricted types of foods, and whether food selectivity impairs functioning. The interviewer had at least a bachelor’s degree in a relevant field in addition to extensive prior experience in working with children and parents. The PAPA includes both required and optional follow-up questions and the administrator decides whether the symptom is present and probes until she or he can make a decision. Based on the interview, the participating children were categorized according to their level of pickiness: no pickiness; moderate pickiness (the child only eats food s/he likes); and severe pickiness (pickiness is substantial and comprehensive, separate meals must be made for the child). Using the same measure of pickiness as in the present inquiry, Zucker et al. (2015) found that even moderate levels of pickiness are associated with psychiatric symptoms and thus need to be identified. We therefore used a dummy variable including both moderate and severe pickiness as the main outcome (0 = no pickiness; 1 = moderate/severe pickiness). Nine percent of videotaped recordings of the PAPA interviews were recoded by blinded interviewers which revealed high inter-rater reliability, ICC = 0.92.

#### Sensory sensitivity

The PAPA [43] was used to capture sensory sensitivity at age 4, assessing seven sensory modes of sensitivity: (1) tactile (e.g. sensitive to special kinds of clothes/fabrics, tags, seams, etc); (2) oral (e.g. sensitive to crisp, hard, soft consistencies); (3) taste; (4) smell; (5) sounds (e.g. sensitive to sharp, loud sounds); (6) visual (e.g. sensitive to bright sunlight); and (7) “other” forms of sensitivity (“sensitive to other kinds/modes of perceptual sensations?”). The interviewer categorized the child as hypersensitive if impairment was reported (e.g. gets emotionally upset, tries to get away from the sensory stimuli) (0 = no sensitivity; 1 = sensitivity). A sum score of the 7 sensory modes was calculated ranging from 0 to 7.

#### Temperament

The Norwegian version of the parent-reported Children’s Behavior Questionnaire (CBQ) long version [22] was used to assess Negative Affectivity (α = .88) and Surgency (α = .92). The CBQ consists of 195 items, rated on a 7-point Likert scale (1 = “Extremely untrue of your child”; 7 = “Extremely true of your child”).

#### Parental sensitivity and structuring

Parent and child interactions were videotaped at T1 during four consecutive 5-min sequences (free play, child lead play, parent led play, and a clean-up task). Parental sensitivity and structuring were rated based on the emotional availability scales (EAS) [44]. Sensitivity captures a parent’s ability to develop and maintain a positive and healthy emotional connection with the child. Highly insensitive parents display few areas of strength in interactions with their child, e.g. only traumatic signals may elicit parental attention, the parent might appear to “forget” that his/her child is around, whereas highly sensitive parents are attentive and responsive (e.g., positive statements, smiling, interest). Structuring refers to the parent’s capacity to support the child’s learning, and an optimally structuring parent teaches or helps the child at the same time as s/he permits a degree of autonomy so that the child can learn independently [28]. Overall assessment of sensitivity and structuring are made across the four sequences using seven subscales for each construct (sensitivity: α = .82; structuring: α = .83). All raters were trained and certified as reliable by Biringen, who developed the EAS-scales. The inter-rater reliability between multiple blinded coders on a random 10% sample of the videotapes was ICC = .62 for both sensitivity and structuring.

#### Socioeconomic status

Socioeconomic status was measured by parental occupation, coded according to the International Classification of Occupations [45] on a 6-point scale (1 = Manual workers, 6 = Leaders). If parents were living together the parent with the highest occupation was chosen.

### Statistical analyses

Logistic regression analyses were used to estimate stability of pickiness from age 4 to 6. In this multivariate model, pickiness at age 6 was regressed on pickiness, temperament, sensory sensitivity and parenting at age 4, allowing predictors to covary. The proposed pathways tested are illustrated in Additional file 1: Figure S1. Because research has shown that low income predicts pickiness [8], analyses were adjusted for parental socioeconomic status. Gender specific analyses were conducted and Wald tests of parameter constraints were used to test if the predictors were different for boys and girls.

Models were performed in Mplus version 7.0 [46]. We applied a robust maximum likelihood estimator, which is robust to moderate deviations from multivariate normality and provides robust standard errors. A full information maximum likelihood procedure was used to handle missing data. This procedure means that analyses are performed on all available data, provided that cases have values for the dependent variable (pickiness) (*n* = 1035). Because we used a screen-stratified sample, all analyses were performed using probability weights, which were the inverse of the drawing probability (i.e. low scorers on the SDQ were weighted up and high scorers were weighted down) to produce accurate population estimates. Analyses revealed that none of the study variables predicted attrition.

## Results

### Preliminary analyses

Preliminary analyses using multinomial logistic regressions between three categories of pickiness (no, moderate and severe pickiness) at age 4 and 6 revealed no difference in predictors of moderate and severe pickiness, supporting the decision to treat pickiness as a dichotomous variable (no versus moderate/severe pickiness).

Table 1 displays the estimated means and SD of all study variables at baseline, as well as the multivariate correlations between the variables. Diagnostic tests to detect multicollinearity issues were run with pickiness at age 6 regressed on pickiness and all predictors at age 4 (children’s negative affectivity, surgency, sensory hypersensitivity, parental structuring and sensitivity) [47]. We did not find any multicollinearity issues with the data (tolerance > .50, VIF < 1.99).

**Table 1:** Means, standard deviations and correlation coefficients between all study variables at baseline

	Mean (SD)	Children’s negative affectivity	Children’s surgency	Children’s sensory sensitivity	Parental sensitivity	Parental structuring	Socio-economic status
Pickiness	0.28 (0.45)	.13***	−.01	.16***	−.02	−.02	−.01
Children’s negative affectivity	3.70 (0.47)		−.18***	.17***	−.08**	−.07*	−.10***
Children’s surgency	3.55 (0.61)			−.04	−.03	−.07*	−.02
Children’s sensory sensitivity	0.23 (0.98)				−.06*	−.05	−.04
Parental sensitivity	25.18 (3.02)					.71***	.15***
Parental structuring	25.69 (3.18)						.13***
Socioeconomic status	4.41 (0.98)						

Biserial correlations are estimated for the dichotomous pickiness variable. *p < .05. **p < .01. *** *p* < .001

### Prevalence and stability of pickiness

At age 4, 25.7% of the boys and 26.1% of the girls were categorized as picky eaters. At age 6, 24.8% of the boys and 26.6% of the girls displayed pickiness. There was no significant difference in the proportions of girls and boys categorized as picky eaters at either measurement point (Age 4: z = .13, *p* = .90; Age 6: z = .55, *p* = .58). Further, as shown in Table 2, there was moderate stability of pickiness from age 4 to age 6. No gender difference in persistence of pickiness was found (Wald *χ* ^*2*^ = 1.49, df = 1, *p* = .22). In the overall sample, 13.9% displayed pickiness at both ages, thus half of those who were picky eaters at age 4 were also categorized as picky eaters two years later.

**Table 2:** Multivariable predictors at age 4 of picky eating at age 6 (*n* = 1035)

	Age 6				
Age 4:	B	95% CI	OR	95% CI	*p*
Pickiness	1.78	1.37, 2.18	5.92	3.95, 8.86	≤.001
Children’s negative affectivity	0.16	−0,29, 0.61	1.17	0.75, 1.84	.485
Children’s surgency	−0.12	−0.45, 0.20	0.88	0.64, 1.22	.455
Children’s sensory sensitivity	0.22	0.04, 0.40	1.25	1.08, 2.23	.019
Parental sensitivity	0.10	0.00, 0.19	1.10	1.00, 1.21	.049
Parental structuring	−0.10	−0.19, −0.01	0.90	0.82, 0.99	.024

The OR’s are adjusted for all other variables

### Predictors of pickiness

Table 2 displays the results of the logistic regression analyses for the whole sample. The model fit information is as follows: Akaike Information Criterion (AIC) = 18,230.69; Bayesian Information Criterion (BIC) = 18,443.21. AIC and BIC are parsimony-adjusted comparative fir indices and models with smaller AIC and BIC are usually considered more parsimonious [48]. As can be seen in the table, higher levels of parenting sensitivity were found to increase the risk for pickiness at age 6, even when pickiness at age 4 was accounted for. Parental structuring predicted comparatively less pickiness at age 6. More sensory sensitive children were at increased risk for persistent pickiness at age 6, whereas temperamental traits did not predict changes in pickiness over time.

Because SES was unrelated to pickiness in the overall model, SES was not included in the multivariate subgroup model and thus the model fit improved (AIC = 15,651.270, BIC = 15,888.447). Wald tests of parameter constraint revealed that the regression slopes from each predictor to pickiness at age 6, accounting for pickiness at age 4, did not significantly differ between genders (Negative affectivity: Wald *Χ* ^*2*^ = 1.53, df = 1, *p* = .212; Surgency: Wald *Χ* ^*2*^ = .88, df = 1, *p* = .35; Sensory sensitivity: Wald *Χ* ^*2*^ = 2.32, df = 1, *p* = .13; Parental sensitivity: Wald *Χ* ^*2*^ = 1.14, df = 1, *p* = .29; Parental structuring: Wald *Χ* ^*2*^ = 1.69, df = 1, *p* = .19).

## Discussion

In light of the high prevalence of picky eating in children and the related negative health outcomes, we aimed to identify predictors of pickiness by following a large and representative sample of Norwegian children from 4 to 6 years of age. Our study adds to existing research by using a semi-structured interview rather than single items or questionnaires thus also capturing impairment of pickiness, and by examining potentially modifiable child and parent predictors. One in four 4 year olds displayed pickiness and the same prevalence was found when they were 6. Pickiness was moderately stable from preschool to school age, and about half of those who displayed pickiness at age 4 were also picky eaters two years later. As expected, our study showed that children who are more sensory sensitive at age 4 are at higher risk for pickiness two years later. As further hypothesized, children who showed high levels of sensory sensitivity and had parents who were high on sensitivity and low on structuring were most likely to display more pickiness over time. Individual differences in temperament did not predict pickiness and there were no gender differences in the prediction of pickiness.

### Sensory sensitivity

This is the first study to show that sensory sensitivity prospectively predicts picky eating, adding to earlier cross-sectional findings. Not only taste and smell sensitivity, but also tactile sensitivity is associated with pickiness in children [49, 50]; we therefore included it in the overall sensory sensitivity variable in our study. It is well known that taste exposure increases acceptance and even liking of rejected food in children [35], but tactile exposure might add to this effect [49], as might visual exposure [51].

### Parental structuring

As hypothesized, parental structuring reduced the risk of children’s picky eating two years later. This result concurs with a previous cross-sectional study examining parental monitoring [52] and a prospective study of parental pressure to eat [53]. Although our study does not reveal the underlying mechanisms, it might be hypothesized that because parents high in structuring facilitate children’s learning and exploration within the child’s zone of proximal development [54], they are better able to systematically promote exposure of unfamiliar and possibly also previously rejected food, thereby challenging the child within the limits of his/her autonomy.

### Parental sensitivity

Although parental sensitivity is a desirable parenting quality associated with a healthy psychosocial development in children (e.g. social competence, emotion regulation) [38], our results indicate that children of highly sensitive parents are at increased odds of future pickiness. Sensitivity captures parents’ physical and emotional responses to children’s signals and communications, emphasizing affective interactions and negotiation of conflict [54]. As hypothesized in our *sensitive pathway* to pickiness, a plausible explanation of the current finding is that highly sensitive parents may accept the child’s reluctance to try new or rejected food and not offer it again, thus reinforcing the child’s pickiness. In contrast, less sensitive parents might not be so responsive to children’s negative reaction to a food, which could potentially promote exposure.

### Gender differences

We did not find gender differences in the stability of pickiness, in contrast to Cano et al. [8] who found boys to be more persistent picky eaters than girls. This inconsistency may be due to different measurement methods (semi-structured interview vs. pickiness operationalized by two items) and length of follow up, but it should be noted that the gender effect detected by Cano et al. [8] was rather small (relative risk ratio [RRR] = .43, *p* = .05). There was also no difference between genders regarding the predictors of pickiness in our study, but gender differences in the persistence of picky eating are worthy of further exploration.

### Limitations

Although the present inquiry has several strengths, such as the longitudinal design, relatively large sample and an interview-based measure of pickiness and sensory sensitivity, some limitations should be noted. Twin studies have shown that pickiness has a strong genetic basis, with >70% of the individual differences in this behavior being accounted for by genetic variation in young children [55]. Although twin studies also show environmental factors to influence interindividual differences in pickiness [21], accounting for genetics may have altered the present results. Because the same instrument (semi-structured parental interview) was used to capture both pickiness and sensory sensitivity, common methods may have inflated the association between the two constructs. In the present study, we adjusted for pickiness at age 4, thereby limiting the common method effect between sensory sensitivity at age 4 and pickiness at age 6. Because the present inquiry is embedded within the larger Trondheim Early Secure Study (TESS), which aims to examine factors related to psychosocial development and development of mental health problems in children, a global measure of parent-child interaction was required and therefore used here. It is plausible that specific parental approaches in response to early signs of picky eating influence the development of pickiness, over and above general aspects of parenting. Future studies should assess specific parent-child interactions around food. To tease out parenting predictors of picky eating future studies should also be powered to examine high vs. low levels of sensitivity and structuring. Since the inter-rater reliability of the parenting variables was moderate, the strengths of the predictions from parenting may be underestimated. It should further be noted that observations of parent-child interactions took place at the University lab, thus reactivity to a novel environment might have affected the results. Finally, to extend the current study, future research should capture longer developmental periods.

### Implications and future directions

Our results suggest that interventions reducing sensory sensitivity or the way it is handled by parents might decrease the risk of picky eating, although clinical trials are needed to test this assumption. One observational study of novel fruit introduction in 2–4 year olds showed that verbal pressure to eat did not affect intake, whereas physical prompting predicted child swallowing and enjoying the new fruit [25]. The authors suggest that moving the fruit towards the child, holding it under the child’s nose or up to the line of sight to encourage smelling, looking at and holding the fruit, increases sensory exposure, which might be the mechanism facilitating acceptance. Thus, encouragement and gentle exposure may work, but there is no indication that enforcement would. Because picky eating varies by age [1], our findings cannot be generalized to other age groups and also needs to be replicated in other cultures.

## Conclusions

We found that highly sensory sensitive children whose parents were high on sensitivity and low on structuring were the ones most likely to display more pickiness over time, even if pickiness seems to be relatively stable between the ages of 4 and 6. It is important to increase the awareness of healthcare providers about the predictors of picky eating, especially those that are modifiable, such as parenting. Although parental sensitivity indeed should be encouraged, sensitive parents may profit from support to increase their adaptive behavior (e.g., expose the child and handle the child’s potential uneasiness) in order to reduce their child’s pickiness. Repeatedly offering unfamiliar and rejected foods in a firm way without pressure while acknowledging child autonomy seems a promising avenue for future research and intervention.

## Additional file

Additional file 1: Figure S1. Graphical representation of the regression model tested. (DOCX 39 kb)

## Abbreviations

AIC: Akaike information criterion
BIC: Bayesian information criterion
CBQ: Children’s behavior questionnaire
EAS: Emotional availability scales
ICC: Intraclass correlation
PAPA: Preschool age psychiatric assessment
SDQ: Strengths and difficulties questionnaire
SES: Socioeconomic status

## References

[1] Dovey TM, Staples PA, Gibson EL, Halford JCG (2008). Food neophobia and ‘picky/fussy’ eating in children: a review. Appetite.

[2] Taylor CM, Wernimont SM, Northstone K, Emmett PM (2015). Picky/fussy eating in children: Review of definitions, assessment, prevalence and dietary intakes. Appetite.

[3] Mascola AJ, Bryson SW, Agras WS (2010). Picky eating during childhood: a longitudinal study to age 11 years. Eat Behav.

[4] Galloway AT, Fiorito L, Lee Y, Birch LL (2005). Parental pressure, dietary patterns, and weight status among girls who are ‘picky eaters. J Am Diet Assoc.

[5] Tharner A, Jansen PW, Kiefte-de Jong JC, Moll HA, van der Ende J, Jaddoe VWV, Hofman A, Tiemeier H, Franco OH. Toward an operative diagnosis of fussy/picky eating: a latent profile approach in a population-based cohort. Int J Behav Nutr Phys Act. 2014;11 10.1186/1479-5868-11-14 PMC3922255 24512388

[6] Jacobi C, Schmitz G, Agras WS (2008). Is picky eating an eating disorder?. Int J Eat Disord.

[7] Zucker N, Copeland W, Franz L, Carpenter K, Keeling L, Angold A, Egger H (2015). Psychological and psychosocial impairment in preschoolers with selective eating. Pediatrics.

[8] Cano SC, Tiemeier H, Van Hoeken D, Tharner A, Jaddoe VWV, Hofman A, Verhulst FC, Hoek HW (2015). Trajectories of picky eating during childhood: a general population study. Int J Eat Disord.

[9] Jacobi C, Agras WS, Bryson S, Hammer LD (2003). Behavioral validation, precursors, and concomitants of picky eating in childhood. J Am Acad Child Adolesc Psychiatry.

[10] Hafstad GS, Abebe DS, Torgersen L, von Soest T (2013). Picky eating in preschool children: The predictive role of the child's temperament and mother's negative affectivity. Eat Behav.

[11] Cano SC, Hoek HW, Bryant-Waugh R (2015). Picky eating: the current state of research. Curr Opin Psychiatry.

[12] Haycraft E, Farrow C, Meyer C, Powell F, Blissett J (2011). Relationships between temperament and eating behaviours in young children. Appetite.

[13] Anzman-Frasca S, Stifter CA, Birch LL (2012). Temperament and childhood obesity risk: a review of the literature. J Dev Behav Pediatr.

[14] Bergmeier H, Skouteris H, Horwood S, Hooley M, Richardson B (2014). Child temperament and maternal predictors of preschool children's eating and body mass index. A prospective study. Appetite.

[15] Ventura AK, Birch LL. Does parenting affect children's eating and weight status? Int J Behav Nutr Phys Act. 2008;5 10.1186/1479-5868-5-15 PMC2276506 18346282

[16] Vollmer RL, Mobley AR (2013). Parenting styles, feeding styles, and their influence on child obesogenic behaviors and body weight. A review. Appetite.

[17] Lucarelli L, Cimino S, D'Olimpio F, Ammaniti M (2013). Feeding disorders of early childhood: An empirical study of diagnostic subtypes. Int J Eat Disord.

[18] Rothbart MK, Derryberry D, Posner MI, JEBTD W (1994). A psychobiological approach to the development of temperament. Temperament: Individual differences at the interface of biology and behavior.

[19] Pliner P, Hobden K (1992). Development of a scale to measure the trait of foof neophobia in humans. Appetite.

[20] Alley TR, Willet KA, Muth ER (2006). Motion sickness history, food neophobia, and sensation seeking. Percept Mot Skills.

[21] Smith A, Herle M, Fildes A, Cooke L, Steinsbekk S, Llewellyn CH: Food fussiness and food neophobia share a common etiology in early childhood. J Child Psychol Psychiatry. 2016;58(2):189–96. 10.1111/jcpp.12647 PMC5298015 27739065

[22] Rothbart MK, Ahadi SA, Hershey KL, Fisher P (2001). Investigations of temperament at three to seven years: the children’s behavior questionnaire. Child Dev.

[23] Rothbart MK, Ahadi SA, Hershey KL (1994). Temperament and social-behavior in childhood. Merrill Palmer Quart.

[24] Dunn W (2001). The sensations of everyday life: Empirical, theoretical, and pragmatic considerations. Am J Occup Ther.

[25] Blissett J, Fogel A (2013). Intrinsic and extrinsic influences on children's acceptance of new foods. Physiol Behav.

[26] Coulthard H, Blissett J (2009). Fruit and vegetable consumption in children and their mothers. Moderating effects of child sensory sensitivity. Appetite.

[27] Farrow CV, Coulthard H (2012). Relationships between sensory sensitivity, anxiety and selective eating in children. Appetite.

[28] Saunders H, Kraus A, Barone L, Biringen Z. Emotional availability: theory, research, and intervention. Front Psychol. 2015;28(6):1069. doi: 10.3389/fpsyg.2015.01069. 10.3389/fpsyg.2015.01069 PMC4516809 26283996

[29] Blissett J (2011). Relationships between parenting style, feeding style and feeding practices and fruit and vegetable consumption in early childhood. Appetite.

[30] Rodenburg G, Kremers SPJ, Oenema A, van de Mheen D. Associations of children’s appetitive traits with weight and dietary behaviours in the context of general parenting. PLoS One. 2012;(12):e50642, doi: 10.1371/journal.pone.0050642. epub 2012 dec. 5. 10.1371/journal.pone.0050642 PMC3515603 23227194

[31] Montano Z, Smith JD, Dishion TJ, Shaw DS, Wilson MN (2015). Longitudinal relations between observed parenting behaviors and dietary quality of meals from ages 2 to 5. Appetite.

[32] Kremers SP, Brug J, de Vries H, Engels R (2003). Parenting style and adolescent fruit consumption. Appetite.

[33] Sleddens EFC, Kremers SPJ, Stafleu A, Dagnelie PC, De Vries NK, Thijs C (2014). Food parenting practices and child dietary behavior. Prospective relations and the moderating role of general parenting. Appetite.

[34] Vaughn AE, Ward DS, Fisher JO, Faith MS, Hughes SO, Kremers SPJ, Musher-Eizenman DR, O'Connor TM, Patrick H, Power TG (2016). Fundamental constructs in food parenting practices: a content map to guide future research. Nutr Rev.

[35] Cooke L (2007). The importance of exposure for healthy eating in childhood: a review. J Hum Nutr Diet.

[36] Remington A, Anez E, Croker H, Wardle J, Cooke L (2012). Increasing food acceptance in the home setting: a randomized controlled trial of parent-administered taste exposure with incentives. Am J Clin Nutr.

[37] Fildes A, van Jaarsveld CHM, Wardle J, Cooke L (2014). Parent-administered exposure to increase children’s vegetable acceptance: a randomized controlled trial. J Acad Nutr Diet.

[38] Biringen Z, Derscheid D, Vliegen N, Closson L, Easterbrooks MA (2014). Emotional availability (EA): Theoretical background, empirical research using the EA Scales, and clinical applications. Dev Rev.

[39] Wichstrom L, Belsky J, Berg-Nielsen TS (2013). Preschool predictors of childhood anxiety disorders: a prospective community study. J Child Psychol Psychiatry.

[40] Goodman R (1997). The strengths and difficulties questionnaire: a research note. J Child Psychol Psychiatry.

[41] Population's level of education [http://www.ssb.no/en/utdanning/statistikker/utniv/aar].

[42] Wichstrøm L, Berg-Nielsen TS, Angold A, Egger HL, Solheim E, Sveen TH (2012). Prevalence of psychiatric disorders in preschoolers. J Child Psychol Psychiatry.

[43] Egger HL, Erkanli A, Keeler G, Potts E, Walter BK, Angold A (2006). Test-retest reliability of the Preschool Age Psychiatric Assessment (PAPA). J Am Acad Child Adolesc Psychiatry.

[44] Biringen, Z. The Emotional Availabiity (EA) Scales Manual, 4th Edn. Boulder: International Center for Excellence in Emotional Availability; 2008.

[45] International Labour Office (1990). ISCO-88 international standard classification of occupation.

[46] Muthèn LK, Muthèn BO: Mplus user’s guide. Sixth Edition. Los Angeles: Muthen & Muthen; 1998–2010.

[47] Cohen J, Cohen P, West SG, Aiken LS (2003). Applied multiple regression/correlation analysis for the behavioral sciences.

[48] Kline R (2016). Principles and practice of structural equation modeling.

[49] Werthmann J, Jansen A, Havermans R, Nederkoorn C, Kremers S, Roefs A (2015). Bits and pieces. Food texture influences food young children. Appetite.

[50] Nederkoorn C, Jansen A, Havermans RC (2015). Feel your food. The influence of tactile sensitivity on picky eating in children. Appetite.

[51] Heath P, Houston-Price C, Kennedy OB (2011). Increasing food familiarity without the tears. A role for visual exposure?. Appetite.

[52] Jani R, Mallan KM, Daniels L (2015). Association between Australian-Indian mothers’ controlling feeding practices and children's appetite traits. Appetite.

[53] Jarman M, Ogden J, Inskip H, Lawrence W, Baird J, Cooper C, Robinson S, Barker M (2015). How do mothers manage their preschool children's eating habits and does this change as children grow older? A longitudinal analysis. Appetite.

[54] Biringen Z (2000). Emotional availability: Conceptualization and research findings. Am J Orthop.

[55] Fildes A, van Jaarsveld CHM, Cooke L, Wardle J, Llewellyn CH (2016). Common genetic architecture underlying young children’s food fussiness and liking for vegetables and fruit. Am J Clin Nutr.

