# Supplementary material for: Child and parent predictors of picky eating from preschool to school age
Source: Int J Behav Nutr Phys Act. 2017 Jul 6;14:87. doi: 10.1186/s12966-017-0542-7 (PMC5498871; doi:10.1186/s12966-017-0542-7)
Supplement: Additional file 1: Figure S1. — Graphical representation of the regression model tested. (DOCX 39 kb) [file 12966_2017_542_MOESM1_ESM.docx]

Additional file 1: Figure S1: Graphical representation of the regression model tested.

Age 4

Age 6

Pickiness

*Note*: Predictors were allowed to covary (not displayed in the figure).

Parental structuring

Childrens’ sensory sensitivity

Parental sensitivity

Childrens’ surgency

Childrens’ negative affectivity

Pickiness
